# The Mechanical and Efficiency Constraints When Swimming Front Crawl with the Aquanex System

**DOI:** 10.2478/hukin-2022-0090

**Published:** 2022-11-08

**Authors:** Catarina C. Santos, Daniel A. Marinho, Mário J. Costa

**Affiliations:** 1 Department of Sport Sciences, University of Beira Interior, Covilhã, Portugal; 2 Department of Sport Sciences, Polytechnic Institute of Guarda, Guarda, Portugal; 3 Research Center in Sports Sciences, Health Sciences and Human Development (CIDESD), Vila Real, Portugal; 4 Centre of Research, Education, Innovation and Intervention in Sport (CIFI2D), Faculty of Sport, University of Porto, Porto, Portugal; 5 Porto Biomechanics Laboratory (LABIOMEP-UP), University of Porto, Porto, Portugal

**Keywords:** propulsive force, direct method, pressure sensors, kinematic, gender, training

## Abstract

The aim of this study was to compare the mechanical and efficiency constraints between free swim and swimming with differential pressure sensors (Aquanex System). These conditions were also analysed to understand the differences between sexes. Thirty young swimmers, 14 boys and 16 girls (12.31 ± 0.67 years) performed three 25-m front crawl maximal bouts under each condition: free swim and swimming with sensors. Under the condition with sensors, swimmers carried the Aquanex System composed of two hand pressure sensors (v.4.1, Model DU2, Type A, Swimming Technology Research, Richmond, VA, USA). The 25-m time (T25) was assessed as a swimming performance variable. The swimming velocity (v), stroke rate (SR), and stroke length (SL) were assessed and calculated as stroke mechanics variables. Thereafter, the stroke index (SI) and arm stroke efficiency (*η*_F_) were estimated for swimming efficiency. Statistical significance was set at p ≤ 0.05. Swimming performance was impaired when swimmers swam with sensors (overall: p = 0.03, d = 0.14; Δ = 1.30%) and a significant decrease in v was found for overall (p = 0.04, d = 0.14; Δ = 1.42%) and the girls’ group (p < 0.01, d = 0.39; Δ = -1.99%). The remaining stroke mechanics variables showed no differences between conditions, as well as for swimming efficiency. Furthermore, there were no differences between girls and boys in free swim and with sensors for all variables. Swimming with the Aquanex System seems not to impose constraints in the mechanics and efficiency of young swimmers, despite differences in swimming performance and v.

## Introduction

The main goal of human competitive swimming is to diminish drag and increase propulsion to achieve a higher swim velocity and, therefore, travel a given distance in the shortest possible time. In this context, an in-depth analysis of key variables is performed regularly to advise swimmers about ways to progress (Barbosa et al., 2021). In the last couple of decades, there has been a boost in technological advances to get a more friendly and ecological assessment in the water. A large set of devices was developed in a diversity of areas, which allowed researchers to carry out a proper assessment of the various factors that influence swimming performance.

One of the recent areas of scientific research includes swimming kinetics (Santos et al., 2021). The ability to produce propulsive force in the water has been a topic of great interest. A differential pressure sensors system (Aquanex System, Swimming Technology Research) was designed to measure swimmers’ propulsive force. This is a user-friendly set-up that allows the swimmer’s displacement throughout the water in a very similar condition to “free swimming” and delivers real-time feedback (Santos et al., 2021). This commercially available hydrodynamic system measures water pressure differences between the palmar/plantar and dorsal surface (Barbosa et al., 2020) of each body limb (i.e., hands and feet), and hence provides force output (N, newton) as the product of pressure and the area.

Previous studies used the Aquanex System to understand the behaviour of propulsive forces generated by the upper and lower limbs during front-crawl (e.g., Barbosa et al., 2020; Morais et al., 2020; Ng et al., 2019) and the butterfly stroke (e.g., Morais et al., 2021; Pereira et al., 2015). Some of them also reported the assessment of kinematic variables while propulsive force was retrieved (e.g., Morais et al., 2021). Although considered accurate, carrying these tiny pressure sensors can impose some mechanical constraints leading to an underestimation or overestimation of kinematic and efficiency data. Since the change of the hand area surface can occur from additional body salience promoted by the sensors, resistive forces, such as pressure drag, can increase and affect arm stroke motion.

The constraints imposed by several devices during underwater testing have already been a topic of interest. Slight changes in the biomechanical pattern have been found when swimmers used the AquaTrainer® snorkel for physiological purposes (Barbosa et al., 2010; Conceição et al., 2013; Ribeiro et al., 2016; Szczepan et al., 2018). However, to date, there is no evidence of whether the Aquanex System impairs the swimming pattern, and what are the constraints derived from using it. This kind of feedback will help researchers and coaches to be comfortable when using this system in their daily tasks.

The aim of this study was twofold: (i) to analyse and compare the mechanical and efficiency constraints between free swim and the Aquanex System; and (ii) to understand if there are differences in response between sexes. It was hypothesised that: (i) swimming with the Aquanex System would impose slight constraints in the front crawl; and (ii) boys and girls would show similar constraints while using the device.

## Methods

### Participants

Thirty young swimmers (14 boys and 16 girls) were recruited to participate in this study (Table 1). Swimmers were assessed at the end of the third macrocycle (peak form) and the inclusion criteria consisted of: (i) being a competitive swimmer; (ii) having at least two years of experience competing in regional or national events; (iii) completing more than four swim training sessions per week; and (iv) not having suffered from any injury in the past six months. Swimmers’ parents or legal guardians were informed about the benefits and experimental risks before signing a written informed consent form. All procedures were in accordance with the Declaration of Helsinki and approved by the Institutional Ethics Committee of the University of Beira Interior (code: CE-UBI-Pj-2020-058).

**Table 1:** Demographics of competitive swimmers.

	Overall (n = 30) M ± 1SD	Boys (n = 14) M ± 1SD	Girls (n = 16) M ± 1SD
Age (years)	12.31 ± 0.67	12.58 ± 0.64	12.07 ± 0.59
Body mass (kg)	48.53 ± 8.43	50.75 ± 7.57	46.62 ± 8.65
Body height (cm)	157.54 ± 7.48	159.63 ± 8.38	155.76 ± 6.06
Arm span (cm)	158.05 ± 8.34	160.82 ± 9.67	155.68 ± 6.06
Dominant upper-limb (cm)	71.02 ± 4.18	72.53 ± 4.54	69.73 ± 3.33
FINA points (50-m freestyle)	270.17 ± 62.27	278.30 ± 75.06	263.92 ± 49.35

kg, kilogram; cm, centimeter.

### Procedures

**Figure 1:**
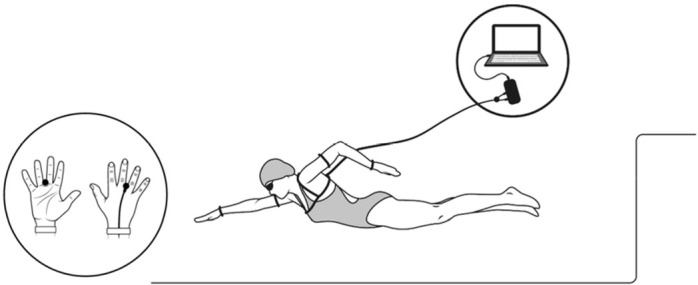
Swimmer carrying the hand differential pressure system with Type A sensors.

The in-water testing took place in a 25-m indoor swimming pool (mean water temperature: 27.5°C) during two consecutive days (24 h apart) in the afternoon period. Swimmers were randomly assigned (first bout) to perform 25-m all-out sprints in front crawl (full stroke), after a standard warm-up previously reported for sprinting events (Neiva et al., 2015). Each swimmer undertook three maximal bouts per each selected condition on separate days: free swim and swimming with sensors. All in-water bouts started by a push-off and swimmers were instructed to maintain their normal breathing pattern for sprinting events. To ensure full recovery, a 30-min rest interval between bouts was applied. All swimmers were encouraged to avoid intense exercise on the data collection days, as well as the day before. The in-water data were assessed in all bouts for both conditions and the best result was considered for further analysis. Under the condition with sensors, swimmers wore a differential pressure system composed of two hand pressure sensors (Type A, Swimming Technology Research, Richmond, VA, USA) positioned between the third and fourth metacarpals (Figure 1).

The shoulders and arms elastic straps allowed the system to be carried during the swimmer's displacement throughout the water and the sensors were connected to an interface connected to a laptop with Aquanex software (v.4.1, Model DU2, Swimming Technology Research, Richmond, VA, USA). The time spent (in s) to cover the predefined distance (i.e., 25 m) was manually assessed by two experts (ICC: 0.97), each with a stopwatch (FINIS 3x100, Finis Inc., USA), and it was considered as a swimming performance variable (T25). The stroke mechanics comprised the swimming velocity (swimming *v*), the stroke rate (SR), and the stroke length (SL). The *v* (in m·s^−1^) was calculated based on the ratio between the distance and T25. The SR (in Hz) was assessed with a chrono-frequency meter (FINIS 3x300, Finis Inc., USA) from three consecutive stroke cycles between the 11^th^ and the 24^th^ m, and the SL (in m) was estimated (SL = *v* / SR) as reported elsewhere (Costa et al., 2020; Craig and Pendergast, 1979). To analyse swimming stroke efficiency, the stroke index (SI, in m^2^·s^−1^) was computed (SI = v · SL) (Costill et al., 1985), and the arm stroke efficiency (η_F_, in %), based on Froude efficiency, was estimated as:


$$\eta_{F}=\left(\frac{v\cdot 0.9}{2\pi \cdot SF\cdot l}\right)\cdot \frac{2}{\pi }$$

in which *l* is the arm’s length (in m) computed as Zamparo et al. (2005) reported.

### Statistical analysis

The normality of the data distribution was checked with the Shapiro-Wilk test. The mean and one standard deviation (M ± 1SD) were computed for all variables, as well as the mean percentage of change (Δ). The dataset for each condition was split into three groups: overall (n = 30), boys (n = 14), and girls (n = 16). The paired sample *t*-test was used to compare both conditions in all variables, whereas the unpaired *t*-test was used to verify the differences between genders (i.e., boys and girls). Cohen’s d was selected as an effect size (d) and interpreted as: trivial if |d| < 0.2, medium if 0.2 > |d| < 0.5, and large if |d| ≥ 0.5 (Cohen, 1988). All statistical analyses were performed in SPSS software (v.27, IBM, SPSS Inc., Chicago, IL, USA) and GraphPad Prism (v.9, GraphPad Software, San Diego, CA, USA). The statistical significance was set at *p* ≤ 0.05.

## Results

**Figure 2:**
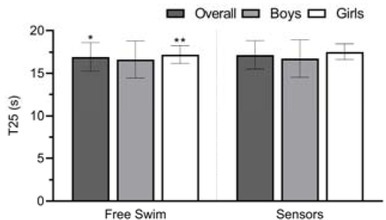
Comparison of swimming performance between free swim and sensors in the front crawl. *p ≤ 0.05 or **p ≤ 0.01, denotes a significant difference to sensors.

The comparison of swimming performance under both conditions is shown in Figure 2. Overall, there was an increase in T25 when swimming with sensors (*p* = 0.03, d = 0.14; Δ = 1.30%), despite the trivial difference. While boys had similar T25 under both conditions (*p* = 0.51, d = 0.05; Δ = 0.61%), girls presented a significant and medium difference (*p* < 0.01, d = 0.35; Δ = 1.90%). The unpaired *t*-test revealed no differences between sexes in T25.

**Figure 3:**
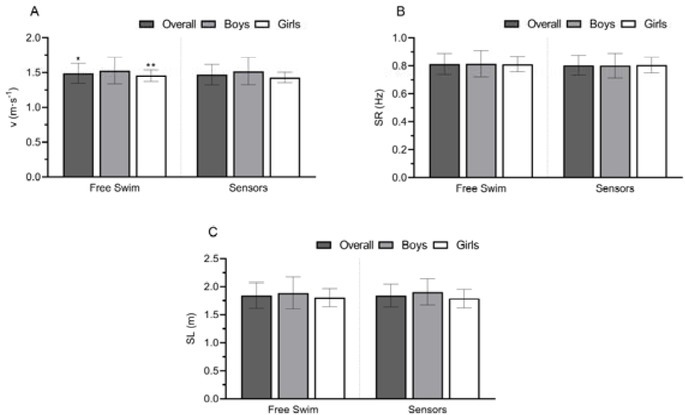
Comparison between free swim and sensors in stroke mechanics variables in the front crawl. Panel A: swimming velocity (v); Panel B: stroke rate (SR); Panel C: stroke length (SL). *p ≤ 0.05 or **p ≤ 0.01, denotes a significant difference to sensors

Figure 3 depicts the comparison between free swim and swimming with sensors according to the stroke mechanics variables. The *v* (Panel A) achieved overall presented a significant although trivial difference (*p* = 0.04, d = 0.14; Δ = 1.42%). Regarding girls, a significant decrease in *v* was found when swimming with sensors (*p* < 0.01, d = 0.39; Δ = -1.99%). The SR (Panel B) and SL (Panel C) were not significantly different between free swim and sensors in all groups. Despite these, Δ in SR decreased by 1.63% and 2.42% with sensors overall and in the boys’ group, respectively, while in the girls’ group Δ in SR increased slightly (Δ = 0.93%). Overall, the SL decreased non-significantly (Δ = - 0.09%), the boys’ SL increased (Δ = 1.09%), and the girls’ SL decreased (Δ = -1.13%). No differences (*p* > 0.05) were also found between sexes in *v*, SR, and SL.

**Figure 4:**
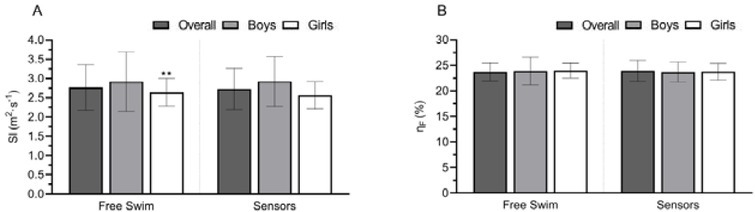
Comparison between free swim and sensors in swimming efficiency variables in the front crawl. Panel A: stroke index (SI); Panel B: arm stroke efficiency (*η*_F_). *p ≤ 0.05 or **p ≤ 0.01, denotes a significant difference to sensors

The swimming efficiency variables are shown in Figure 4. There was a significant decrease in the girls’ SI (Panel A) with sensors (*p* = 0.01, d = 0.20; Δ = -3.15%). The Δ was -1.53% and 0.32% overall and in the boys’ group, respectively. No differences were found in η_F_ (*p* > 0.05) for all groups. However, there was a slight tendency to decrease η_F_ when swimming with sensors (overall, Δ = -0.90%; boys, Δ = -0.79%; girls, Δ = -1.01%). The sex comparison revealed no differences (*p* > 0.05) in SI and η_F_.

## Discussion

This study considered the technical constraints induced by the Aquanex System when swimming front crawl. The main finding was that swimming with sensors imposed trivial constraints on swimming performance and *v* but did not change the stroke mechanics or efficiency of young swimmers. Trivial constraints appeared to be more related to the girl’s cohort. Nevertheless, there were no differences between sexes under both conditions for all variables.

Front crawl has been recognised as the fastest and most economical swimming stroke (Barbosa et al., 2010; Deschodt et al., 1999), being the most reported for field-oriented research purposes and for tracking swimming performance. Sprint events in short- and long-course swimming pools are characterised by generating a greater amount of propulsion in the water to reach higher velocity (Seifert et al., 2007). Thus, this kind of assessment is crucial and needs to be as accurate as possible, imposing the least constraints in the various aspects of the stroke.

Overall, front crawl swimming performance decreased significantly (1.30%) by adding the sensors (i.e., T25 increase), and thereby the *v* decreased as well by 1.42% during the T25. The *v* is highly dependent on the interaction between propulsive and resistive forces (Toussaint and Truijens, 2005). In front crawl, the upper limbs have been described as the most responsible for propulsion (Barbosa et al., 2020; Deschodt et al., 1999). As the system is carried by elastic straps in the upper limbs, swimmers might be under an additional drag. Likewise, changes in the palmar surface area due to the pressure sensors may also increase resistive forces (Santos et al., 2021). Previous studies using an additional device in the water (e.g., AquaTrainer® snorkel) have reported a similar decrease in swimming performance and *v* during front crawl and breaststroke (Barbosa et al., 2010; Conceição et al., 2013). The same authors argue that the decrease in *v* when adding the device, and therefore in the testing time, may be related to the existent passive and active drag.

Another important aspect is how the all-out effort was performed. Swimmers were assessed in a short distance (i.e., 25 m) with an in-water start. This was performed equally under both conditions without diving and adding a dolphin kick. When using sensors, it can be argued that the decrease found in swimming performance and *v* can be derived from a slower start as swimmers may need an initial adjustment to their swimming pattern. This may help explain the differences in the testing time (i.e., T25) between free swimming and swimming with sensors, but it does not impair the related mechanical aspects of the stroke.

The SF was assessed considering the 11^th^ and the 24^th^ m of the pool. It seems that swimmers were able to maintain their motion with and without the system. Theoretically, *v* can be modified by an increase or a decrease of the SR and SL (Barbosa et al., 2011). The results showed that the SF and the SL were not significantly different between both conditions in all groups, despite the differences previously found in *v*. Probably, the above-mentioned adaptation to the system (cable plus hand’s set-up) after the start happens until the 11^th^ m, not affecting both the SR and the SL measured afterwards. The same trend may be observed in efficiency. Since swimming efficiency was estimated based on stroke mechanics and/or anthropometric features, the SI and 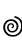
_F_ were similar under both conditions for the pooled sample (i.e., overall group). Normally, the stroke mechanics variables, including the SR and the SL, and, therefore, the efficiency are dependent on limbs kinematics (Barbosa et al., 2010; Barbosa et al., 2011). Within this rationale, limbs trajectories and velocities may be decreased when using larger (Gourgoulis et al., 2006) and resistive (Guignard et al., 2017) devices. The system used in this study consisted of two small lightweight sensors attached to the swimmers’ hands. Although it is considered an external device, it seems not to promote sufficient fatigue or increase resistive forces to change limb kinematics and stroke mechanics.

Boys and girls were analysed together at a first stage, since the sex gap is not an issue in this age group, at least with regard to pre-adolescence (Seifert et al., 2011; Zuniga et al., 2011). However, this does not mean that, at some point, the behaviour between boys and girls will not be interpreted separately (Barbosa et al., 2014). Within this approach, while girls showed decreases in *v* and SI when using the sensors, boys were not as constrained as girls. Explanations may rely on the boys’ better ability for power output (Barbosa et al., 2015), which may enable them to adapt and sustain their effort even when using external devices. Although the sex comparison was performed, no differences were noted for all variables.

We may point out few limitations in the present research: (i) the *v* assessment was conducted based on T25 and distance (25 m), instead considering the range between the 11^th^ and the 24^th^ m used for the remaining variables; and (ii) the assessment of kinematics and timing should rely on cutting edge set-ups (e.g., high velocity cameras or phototiming) to get even a more precise measurement.

## Conclusion

The Aquanex System seems not to induce constraints on the mechanics and efficiency of young swimmers, which can allow coaches to use it in their daily practice for monitoring of the training process. Despite that, coaches and researchers are advised to take some care in its application because during all-out efforts the initial velocity of the test can be compromised. As the cable can be an issue, a necessary quick adaptation to the device after the start is needed. As such, this can slightly compromise the mean velocity if we consider the overall distance covered for velocity estimation. Thus, measures such as swimming velocity, mechanics of the stroke, and efficiency, along with propulsive force should be retrieved further in the test for a more accurate assessment.
